# An Unusual and Unexpected Result of Diagnostic Sampling for COVID-19

**DOI:** 10.7759/cureus.10979

**Published:** 2020-10-16

**Authors:** Mousa S Hussein, Mansoor Hameed, Mona Allangawi, Hisham Abdelsattar, Irfan Ul Haq

**Affiliations:** 1 Pulmonology, Hamad Medical Corporation, Doha, QAT; 2 Medicine, Weill Cornell Medicine, Doha, QAT

**Keywords:** covid 19, pandemic, swab, foreign body, tracheostomy

## Abstract

Coronavirus disease 2019 (COVID-19) is an infectious disease caused by severe acute respiratory syndrome coronavirus 2 (SARS-CoV-2). It has spread globally, resulting in an ongoing pandemic. Real-time reverse transcription-polymerase chain reaction (rRT-PCR) from a viral swab is diagnostic. The most common site to take this swab is from the nasopharyngeal area; however, patients with tracheostomies represent a major challenge as they have two sources for colonization and possible infection including the nose and the trachea. We present the case of a patient who had a COVID-19 diagnostic swab through his tracheostomy, when unfortunately the swab broke, resulting in a bronchial foreign body.

## Introduction

Coronaviruses are viral pathogens causing various manifestations of respiratory diseases in humans, ranging from the common cold to the sometimes fatal infections from severe acute respiratory syndrome coronavirus (SARS-CoV) and the Middle East respiratory syndrome coronavirus (MERS-CoV) which are of zoonotic origin. Currently, we are facing a pandemic caused by the severe acute respiratory syndrome coronavirus 2 (SARS-CoV-2) which is a single-stranded RNA virus belonging to the family of coronaviruses. The spread is believed to have started initially in Wuhan, China at the end of 2019, spreading throughout the world since then [1]. The key step in the diagnosis of this disease, along with a high clinical index of suspicion based on the most common symptoms of fever, cough, shortness of breath, and loss of taste and smell, is the use of real-time reverse transcription-polymerase chain reaction (rRT-PCR) from respiratory tract samples [2]. Early testing can result in rapid detection and effective isolation, hence, limiting transmission [3]. 

## Case presentation

A 98-year-old male patient, with a background of dementia, previous stroke with persistent generalized weakness, and a permanent tracheostomy on long term ventilation was transferred to our tertiary care center.

**Figure 1:**
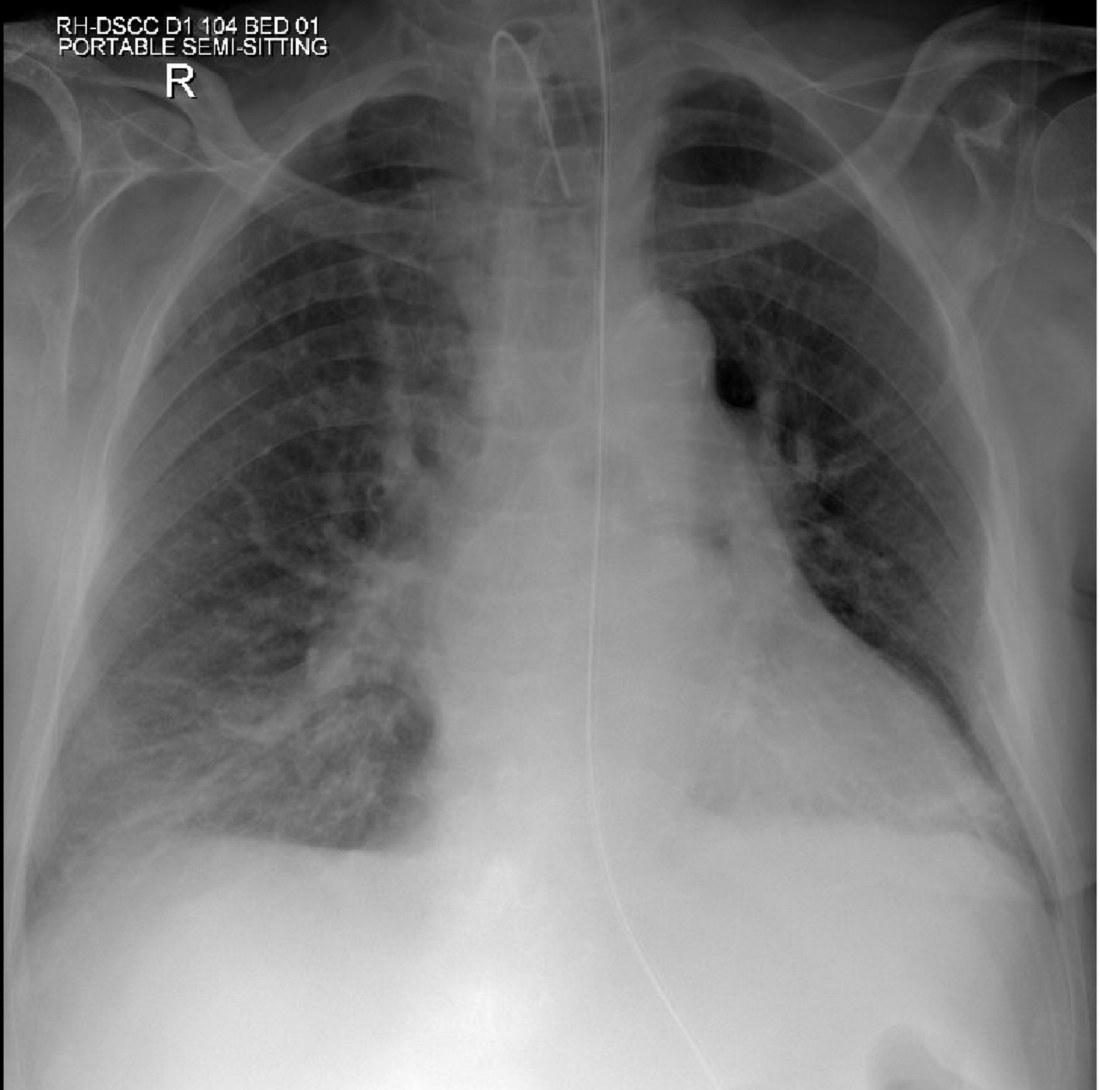
Chest X-ray showing nasogastric tube and tracheostomy tube with no clear evidence of foreign body (radiolucent)

As part of his long-term care facility's policy, he had a coronavirus disease 2019 (COVID-19) tracheal swab for screening. He did not have any exposure or symptoms of COVID-19. During the process of obtaining the viral swab from the trachea through the tracheostomy tube, unfortunately, the swab stick broke and fell into the trachea inducing cough, agitation, and restlessness in the patient. An immediate chest X-ray was unremarkable (Figure 1). 

**Figure 2:**
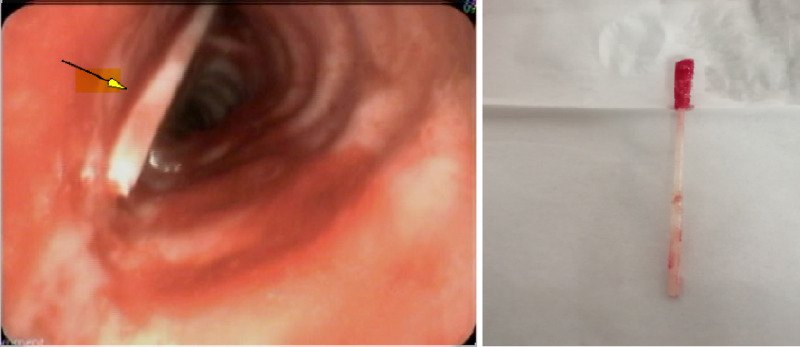
Foreign body (swab stick) impacted near the entrance of the left upper lobe

The patient’s care facility team immediately involved otorhinolaryngology. Fibro-optic examination by them, through the tracheostomy, showed the broken part of the swab in the left main bronchus. The patient was then shifted to our tertiary care center whereby a bronchoscopy was performed, which confirmed the broken swab stick in the left main bronchus. The swab stick was retrieved with a toothed grasping forceps and taken out through the tracheostomy tube. The retrieved portion was about 5 cm long (Figure 2). 

## Discussion

COVID-19 disease can affect people of all ages; however, it has resulted in a more severe course in patients older than 60 or with chronic conditions such as chronic lung disease, coronary artery disease, diabetes mellitus, hypertension, and those who are immunocompromised. Most of the residents in long-term care facilities, are not only of older age but also have at least one of these chronic conditions putting them at a high risk of severe disease [4]. Their risk, therefore, puts them at priority to be routinely screened and tested for COVID-19 disease. No screening test is, however, perfect and the same is true for COVID-19, hence the results of screening need to be interpreted in the context of pre-test probabilities and clinicoradiological characteristics [5].

The causative organism for COVID-19 disease is the SARS-CoV-2 virus which predominantly affects the respiratory epithelium that extends from the nose down to the smallest respiratory units, with a higher viral load usually found in the nasopharynx [6]. The most widely used method to confirm the diagnosis of SARS-CoV-2 infection is via molecular testing using rRT-PCR to detect the viral RNA. The usual way to take viral samples is through nasopharyngeal swabs from the respiratory epithelium lining the nasopharynx. Other methods of sampling include sputum samples, bronchoalveolar lavage, or a bronchoscopic brush biopsy with a variable degree of accuracy [7]. One of the serious reported complications of taking a swab is that of the swab stick breaking, resulting in the broken part getting stuck as a foreign body in the nasopharynx or the lower respiratory tracts [8,9].

Patients with a tracheostomy present a challenge in terms of testing for SARS-CoV-2 as they have altered anatomy. Therefore, they may have two potential sites of colonization/infection with SARS CoV-2. Nasopharyngeal colonization even if no airflow is occurring through the nose or tracheal colonization through either hand contamination/touching as is required during phonation and routine tracheostomy care, or from aerosolized exposure.

Thw American Head and Neck Society (AHNS) recommends tracheal swabs as part of initial diagnostic testing for COVID-19 in patients with pre-existing tracheostomies [10]. This is in line with the Center for Disease Control (CDC) recommendations of the upper respiratory tract, and lower respiratory tract specimens, if available [11].

Extra caution should be taken in these already likely frail patients with multiple comorbidities, during sampling to avoid local injury or tracheobronchial foreign bodies. Other measures to avoid such complications include the use of the newer type of swabs without a breakpoint that has been approved by the World Health Organization (WHO) for clinical use. An experienced member of staff should perform the swab in such patients, and it also helps to ensure that the patient is in a calm and relaxed state; as any agitation or unpredicted movements while swabbing can increase the risk of the swab breaking [12].

## Conclusions

Infection prevention and control practices during the COVID-19 pandemic in long term care/assisted living facilities catering to patients may require COVID-19 screening as part of local policies, to identify those with asymptomatic or pre-symptomatic SARS-CoV-2 infection. Full safety measures and extra cautions should be taken while taking samples from these patients to avoid any complications. There is also a need to have a clear policy and guidance on how to test patients with tracheostomies for SARS-CoV-2. To our knowledge, this is the second reported case of a bronchial foreign body due to a broken COVID-19 viral swab stick in a tracheostomized patient.
